# The Effect of Intrathecal Bupivacaine Plus Dextrose 5% and Fentanyl Compared with Bupivacaine Alone on the Onset and Duration of Analgesia in Patients Undergoing Lower-Limb Orthopedic Surgery

**DOI:** 10.1155/2023/2496557

**Published:** 2023-02-14

**Authors:** Amir Sabertanha, Gholam Reza Makhmalbaf, Maryam Bayati, Aram Meshkini

**Affiliations:** ^1^Department of Anesthesiology, School of Medicine, Birjand University of Medical Sciences, Birjand, Iran; ^2^Department of Anesthesiology, Birjand University of Medical Sciences, Birjand, Iran

## Abstract

**Introduction:**

This study aimed to compare the effect of intrathecal bupivacaine plus dextrose 5% and fentanyl with bupivacaine alone on the onset and duration of analgesia in patients undergoing lower-limb orthopedic surgery.

**Materials and Methods:**

A total of 40 patients eligible for lower-limb surgery were divided into two groups by simple randomization: the control group which received only bupivacaine and the intervention group which received bupivacaine plus dextrose 5% and fentanyl. Anesthesia was induced by the spinal method. The visual analog scale (VAS) was used to assess the patients' pain; hemodynamic status (systolic and diastolic blood pressure and the heart rate) and oxygen saturation were also monitored.

**Results:**

There was a significant difference between groups in the type of lower-limb movement at the L1 anesthesia level, the sensory block level at time zero after surgery, the type of backward movement at time zero after surgery, and the analgesic dose received (*p* < 0.05). Fifteen and 30 minutes after the start of surgery, mean systolic blood pressure, and 45 and 60 minutes after the start of surgery, systolic and diastolic blood pressure and the heart rate were significantly lower in the control group than in the intervention group (*p* < 0.05). The VAS score was significantly lower in the intervention group than in the control group at 6 and 24 hours after surgery (*p* < 0.05). Systolic and diastolic blood pressure at time zero, systolic blood pressure at hour 6, and diastolic blood pressure at hour 24 after surgery were significantly lower in the control group than in the intervention group (*p* < 0.05).

**Conclusion:**

The mean duration of anesthesia and analgesia was significantly longer in patients receiving bupivacaine plus fentanyl than in those receiving bupivacaine alone. However, concerning hemodynamic parameters, it cannot be concluded that the bupivacaine plus fentanyl receiving group was generally superior to the bupivacaine receiving group.

## 1. Introduction

Pain of varying intensity is inevitable after any surgery. According to studies, 80% of patients suffer moderate to severe postoperative pain [1, 2].

Persistent pain can lead to hemodynamic instability, unfavorable surgical outcomes, patient dissatisfaction, an increased length of stay, and increased patient costs [3–6].

Many factors play a role in the development of postoperative pain, such as patient tolerance, anesthetic method, postoperative analgesics, and surgical procedure [7, 8]. Various scales are used to assess pain, including the verbal pain scale (VPS), descriptor differential scale (DDS), numerical rating scale (NRS), visual analog scale (VAS), and faces pain scale (FPS). The diagnosis and assessment of pain should be performed systematically and continuously. In addition, the contribution of treatment to pain relief should also be reported [9]. Pain relief in hospitalized patients reduces the length of stay, nosocomial infections, and hospitalization costs [10, 11].

Disadvantages of general anesthesia include nausea, vomiting, shivering, agitation, pulmonary complications, and an increased bleeding rate. On the other hand, regional anesthesia (spinal anesthesia) has some advantages over general anesthesia, such as lower pulmonary complications, a lower bleeding rate, and adequate analgesia, resulting in a better patient condition [12, 13].

Drugs used for anesthesia include bupivacaine, lidocaine, tetracaine, and ropivacaine (14). Bupivacaine is the most commonly used local anesthetic for regional anesthesia, especially for peripheral nerve blocks [14].

Bupivacaine is a long-acting anesthetic commonly used in regional anesthesia for pain management during surgery. It is also commonly used for peripheral nerve blocks and has an impeccable history in spinal anesthesia [14].

Fentanyl is an opioid used to induce and maintain anesthesia during general anesthesia [15]. It is highly lipid-soluble and binds to proteins. Fentanyl and its derivatives have pharmacological effects on µ-opioid receptors [16]. Respiratory disturbance is the most common complication of fentanyl. Other complications include nausea, vomiting, constipation, pruritus, drug dependence, bradycardia, and skeletal muscle rigidity. Hemodynamic changes may rarely occur after anesthesia [17].

Pain control is one of the most important challenges during and after surgery. Therefore, prevention and relief of pain after surgery are the main concerns of surgical departments and play a crucial role in improving the general condition of patients in the hospital. On the other hand, the use of anesthetics, including bupivacaine, is associated with some complications such as hemodynamic and cardiac complications, which are dose-dependent and can be reduced by decreasing the drug dose (17). In this study, we reduced the dose of bupivacaine and added other drugs for regional anesthesia to reduce the adverse effects of bupivacaine on hemodynamics and achieve a stable analgesic effect during and after surgery in patients. This study aimed to compare the effect of intrathecal bupivacaine plus dextrose 5% and fentanyl with bupivacaine alone on the onset and duration of analgesia in patients undergoing lower-limb orthopedic surgery.

## 2. Methodology

This randomized double-blind clinical trial was approved by the Ethics Committee of Birjand Medical Sciences University under the code IR.BUMS.REC.1398.351. The study was also registered on the Iranian clinical trials website under the code IRCT20190618043934N2.

All patients (or their companions) signed informed consent to participate in the study. Refusal to participate in the study did not affect their course of treatment. The study population included all patients eligible for lower-limb surgery. Inclusion criteria were patients in American Society of Anesthesiologists (ASA) classes 1 and 2, age between 20 and 50 years, no drug dependence, informed consent, no spinal surgery in the last week, no pregnancy, no recent traumatic brain injury, and no spinal deformity.

Exclusion criteria were patient withdrawal, surgery duration longer than 1 hour, patient inability to position, spinal infection, sensitivity to local anesthetics, high ICP, and coagulation disorders.

The American Society of Anesthesiologists (ASA) classifies patients undergoing surgery into 6 groups based on their physical condition, regardless of the surgical procedure. This classification provides a general description of the patient's status. The ASA classification is as follows:

  ASAI: normal, healthy patients with no systemic problems such as cardiac, vascular, respiratory, or endocrine disease, e.g., a healthy person scheduled for surgery.
  ASAII: patients with a mild systemic disease in which the disease is controlled and does not limit the patient, e.g., controlled hypertension, controlled diabetes, chronic bronchitis, and obesity.
  ASAIII: patients with a severe systemic disease that imposes functional limitations, e.g., uncontrolled hypertension, complicated diabetes, coronary artery disease, and thyrotoxicosis.
  ASAIV: patients with severe systemic diseases that endanger their lives, e.g., congestive heart failure, unstable angina, advanced pulmonary, renal, or hepatic dysfunction.
  ASAV: moribund patients in whom survival without surgery is unlikely, e.g., ruptured abdominal aneurysm, pulmonary embolism, and head trauma with increased intracranial pressure.
  ASAVI: declared brain-dead patients undergoing surgery for organ donation.

### 2.1. Sample Size and Sampling

The sample size was estimated based on the study by Bezai et al. [18] and the following formula. The mean and standard deviation of the analgesia score 15 minutes after surgery were as follows. The sample size was calculated to be 18 patients for each group using the following formula. Considering an attrition rate of 10%, the sample size was increased to 20 patients for each group. Thus, a total of 40 patients were included in this study.

(1) $$\begin{array}{c} n=\frac{{\left(S_{1}^{2}+S_{2}^{2}\right)\left(Z_{1-\alpha /2}+Z_{1-\beta }\right)}^{2}}{{\left(\mu_{1}-\mu_{2}\right)}^{2}}\text{,}\\ Z_{1-\alpha /2}=\frac{1}{96}\text{,}\\ \alpha =0.05\text{,}\\ Z_{1-\beta }=0.84\text{,}\\ \beta =0.2\text{,}\\ S_{1}=8.8\text{,}\\ S_{2}=11.2\text{,}\\ \mu_{1}=110\text{,}\\ \mu_{2}=102.8\text{.} \end{array}$$

### 2.2. Sampling

The participants were selected from patients undergoing lower-limb surgery and divided into two 20-patient groups of control and intervention by the sample randomization and blocking method (four blocks); one group received 2 mL (10 mg) of marcaine 0.5% (A or control), and the other received 1 mL (5 mg) of marcaine 0.5% plus 0.5 mL (25 µg) of fentanyl and 0.5 mL of sterile dextrose 5% (B or intervention). First, various four-blocks were developed (AABB, BBAA, ABAB, BABA, ABBA, and BAAB); then, one block was randomly selected, and based on the order of the selected block, patients were included in one of the groups A or B. Randomization was then performed similarly for other patients.

### 2.3. Intervention

All included patients were fasted for 8 hours and did not receive any analgesics 6 hours before surgery. The VAS was trained for all participants. Patients were placed in the study groups after entering the operating room based on the described randomization method. Neither the patient nor the assessor was aware of the study group.

In the operating room, 200 mL of normal saline was infused before induction of spinal anesthesia, and the pain intensity was assessed with the VAS. Vital signs were also checked. Spinal anesthesia was induced using a Quinke needle G26 at the injection rate of 0.2 mL/s. Immediately after injection, patients were positioned in the Trendelenburg position, and after 30 seconds, their anesthesia level was checked every 30 seconds by the Pin Prick test. After anesthesia reached the L1 level, patients were returned to the supine position, and their ability to move their healthy foot was evaluated; then, they received 2 mg of midazolam. A tourniquet was applied, and all patients were oxygenated during surgery with a mask at a rate of 4–6 L/min.

Vital signs were checked every 5 minutes during the first 15 minutes. Patients with over 20% drop in blood pressure and a heart rate of <50 received inotrope. Those with nausea received atropine or androsterone. Shivering during surgery was treated with 25 mg of pethidine or androsterone.

The onset of anesthesia is as follows: the time interval between induction of anesthesia and anesthesia at L1 and the time interval between anesthesia at L1 and anesthesia at two levels below were measured.

### 2.4. Study Variables

After surgery, a trained nurse who was unaware of the surgery type and the drug received assessed pain intensity with the VAS, the anesthesia block level with the Pin Prick test, movement ability with the Bromage scale, and vital signs at time zero (entering of the patient to the recovery room) and 6, 12, and 24 hours later. The obtained data were recorded in a form. Patients with pain intensity of category 4 received 1 mg of morphine with an interval of at least 30 minutes. The time interval between anesthesia at the desired level and the analgesic injection was considered the effective analgesia time. The opioid doses received by patients in the first 24 hours were recorded.

The hemodynamic parameters (systolic and diastolic blood pressure and the heart rate) and oxygen saturation of the patients during surgery, recorded by monitors in the operating room, were checked.

### 2.5. Data Analysis

The data obtained were analyzed with SPSS-18 using the independent *t*-test or Mann–Whitney *U* test, the paired *t*-test or Wilcoxon test, the Friedman test, and repeated-measures analysis of variance for quantitative variables and the Chi-square or Fischer's exact test for qualitative variables at the significance level of *α* = 0.05.

## 3. Results

This study was carried out on 40 patients in two groups: one received bupivacaine, and the other received bupivacaine plus dextrose 5% and fentanyl. The results showed no significant difference between the groups in sex frequency distribution and the mean age and height of patients (*p* > 0.05).

However, a significant difference existed between the groups in the type of lower-limb movement at the L1 anesthesia level, the anesthesia block level at time zero after surgery, the type of backward movement at time zero after surgery, and the analgesic received (*p* < 0.05) (Table 1).

As shown in Table 2, there was no significant difference between the groups in the frequency distribution of spinal anesthesia complications and the drugs administered (*p*=0.206).

According to the results, no significant difference existed between the groups in the mean VAS score at time zero and 12 hours after surgery (*p* > 0.05). The mean VAS score was significantly lower in the intervention group than in controls at 6 and 24 hours after surgery (*p* < 0.05). Based on the Friedman test, there was a significant difference in the mean pain score at different times studied (*p* < 0.05). The Wilcoxon test showed that the mean VAS score at time zero after surgery was significantly higher than the mean VAS score at hours 6 (*p* < 0.001), 12 (*p*=0.002), and 24 (*p* < 0.001). In addition, the mean VAS score in the intervention group was significantly higher 24 hours after surgery compared to 6 hours after surgery (*p*=0.006).

Table 3 shows no significant difference between the intervention and control groups in systolic blood pressure at time zero and 5 and 10 minutes and 12 hours after surgery (*p* > 0.05). The mean systolic blood pressure was significantly lower in the control group than in the intervention group at time zero, 15 and 30 minutes, and at 6 and 24 hours after surgery (*p* < 0.05).

As shown in Table 4, no significant difference existed between the groups in the mean diastolic blood pressure at time zero, 5, 10, 15, and 30 minutes, and 6, 12, and 24 hours after surgery (*p* > 0.05). The mean diastolic blood pressure was significantly lower in the control group than in the intervention group at time zero and 45 and 60 minutes after surgery (*p* < 0.05). According to the results of repeated-measures ANOVA, there was a significant difference in the mean diastolic blood pressure at different times studied (*p* < 0.001).

No significant difference existed between the groups in the mean heart rate at time zero, 5, 10, 15, and 30 minutes, and at 6, 12, and 24 hours after surgery (*p* > 0.05) (Table 5). The mean heart rate was significantly lower in the control group than in the intervention group at 45 and 60 minutes after surgery (*p* < 0.05). According to the results of repeated-measures ANOVA, there was a significant difference in the mean heart rate at different times studied (*p* < 0.001).

## 4. Discussion

There was a significant difference between the groups in the type of lower-limb movement at the anesthesia level, the anesthesia block level, and the type of backward movement at time zero and the analgesic dose received (*p* < 0.05).The analgesic dose was lower in the bupivacaine receiving group compared to the bupivacaine plus fentanyl receiving group.

Kim et al. showed no significant difference in the mean depth of the anesthesia block level between the bupivacaine plus fentanyl receiving group and the bupivacaine plus sufentanil receiving group [15]. This study is inconsistent with ours, which can be attributed to the difference in surgery type, groups studied, and drugs injected.

In a study by Ferrarezi et al., there was no significant difference in the movement block level and the anesthesia block level between different groups receiving fentanyl and bupivacaine (*p* > 0.05) [16]. This study is not in line with ours, which can result from the difference in drug dose and surgery type.

Gupta et al.stated that the bupivacaine group and the bupivacaine plus fentanyl group had no significant difference in the anesthesia block level of the upper limb and the reverse time of the anesthesia level (*p* > 0.05) [17]. This study is inconsistent with ours due to differences in surgery type, drug dose, and anesthesia method.

In a study by Imani et al.performed on patients undergoing knee arthroscopy, the use of analgesics was reduced in patients receiving bupivacaine plus pethidine (opioid agonist) compared to those receiving only bupivacaine or pethidine [19]; this study is consistent with ours.

Jafarzadeh et al.showed no significant difference between the two groups in the anesthesia time (*p* > 0.05) and the anesthesia block level, and the movement block reversal time was significantly lower in the bupivacaine alone group (*p* < 0.05) [20], which is in line with our study.

Bagherzadeh et al. [21] stated that the movement block was complete in the control group and incomplete in the intervention group; this is inconsistent with our study, where the sensory block was incomplete in the control group and complete in the control group.

This inconsistency may be attributed to the type and the dose of anesthetic used. The dose of analgesic was significantly lower in the bupivacaine plus fentanyl group than in the bupivacaine alone group; this is in line with our study [18]. In addition, the sensory block level was significantly higher in the control group than in the intervention group, which is consistent with our study.

The mean time of anesthesia and analgesia onset was significantly lower in the control group than in the intervention group (*p* < 0.001), and the mean time of anesthesia was significantly lower in the intervention group than in the control group (*p* < 0.001). There was no significant difference between the groups in the mean systolic and diastolic blood pressure and the heart rate at times zero, 5 and 10 minutes after surgery (*p* > 0.05). The mean systolic blood pressure at 15 and 30 minutes after surgery and systolic and diastolic blood pressure and the heart rate at 45 and 60 minutes after surgery were significantly lower in the control group than in the intervention group (*p* < 0.05). In addition, there was no significant difference between the groups in diastolic blood pressure and the heart rate at 15 and 30 minutes after surgery (*p* > 0.05).

In the study of Jabal Ameli et al. [22], adding pethidine to bupivacaine and fentanyl did not result in a significant difference between the study groups in the mean heart rate and systolic and diastolic blood pressure. This study is consistent with ours in some studied times and inconsistent in some other times [20]. The inconsistency may result from the difference in the disease type, drugs, and their dose.

Harssor and Vikram showed no significant difference between the control (bupivacaine) and intervention (bupivacaine and fentanyl) groups in hemodynamic changes [23].

This study is consistent with ours in certain times and inconsistent in some others [20]. The inconsistency can be attributed to the differences in the disease type, the dose of drugs, and the sex of patients (the study of Zirak was performed on pregnant women, whereas our study was carried out mainly on men). Zirak et al. showed no significant difference in the mean anesthesia time and the sensory block reversal time between the control and intervention groups, which is inconsistent with our study. This inconsistency may result from the difference in surgery and the doses of drugs [24].

There was no significant difference in the mean VAS score at time zero and 12 hours after surgery, diastolic blood pressure and the heart rate at 6 and 24 hours after surgery, systolic and diastolic blood pressure and the heart rate at 12 hours after surgery, and the heart rate at time zero after surgery (*p* > 0.05). The mean VAS score at 6 and 24 hours after surgery was significantly lower in the intervention group than in the control group (*p* < 0.05). Systolic and diastolic blood pressure at time zero and systolic blood pressure at 6 and 24 hours after surgery were significantly lower in the control group than in the intervention group (*p* < 0.05).

In a study by Gupta et al., there was no significant difference between the bupivacaine plus fentanyl and bupivacaine groups in the pain score one week after hernia surgery (*p* > 0.05), which is not in line with our study [17]. This inconsistency may result from the difference in surgery type, the time of pain assessment, and sex distribution.

There are various analgesia regimens, including intrathecal injection of opioids alone or in combination with local anesthetics. Combining an opioid with a local anesthetic can reduce their dose without affecting the quality or duration of analgesia. In addition, the drug's adverse effects will also be decreased [25].

## 5. Conclusion

The results of this study showed that the mean time of anesthesia onset and the analgesia duration were significantly longer in the bupivacaine plus fentanyl group than in the bupivacaine alone group (*p* < 0.05). However, concerning hemodynamic parameters, it cannot be concluded that the group receiving bupivacaine plus fentanyl was generally superior to the group receiving bupivacaine alone.

## Figures and Tables

**Table 1:** Comparison of frequency distribution of the movement type, sensory block and painkiller dose.

	Control mean ± SD	Intervention mean ± SD	
Duration of onset of anesthesia	2.8 ± 9.119	7.6 ± 5.156	*t* = 15.434
			*p* < 0.001

Duration of pain relief	1.21 ± 8.194	8.20 ± 4.247	*t* = 7.918
			*p* < 0.001

Duration of numbness	5.15 ± 151	9 ± 1.104	*t* = 11.636
			*p* < 0.001

**Table 2:** Comparison of side effects and prescription drugs.

Study group time		Control	Intervention	
Complication	Vomiting	(66.7) 2	(33.7) 1	*p*=4.512
	Blood pressure	(66.7) 5	(33.7) 2	df = 3
	Bradycardia	(100) 2	(0) 0	*p*=0.206

Type of drug	Ondansetron	(66.7) 2	(33.7) 1	*p*=4.512
	Ephedrine	(66.7) 5	(33.7) 2	df = 3
	Atropine	(100) 2	(0) 0	*p*=0.206

**Table 3:** Comparison of mean systolic blood pressure of two groups at different times.

	Study group time		
	Control mean ± SD	Intervention mean ± SD	
Primary	136.8 ± 11.6^b^	136 ± 1.13	*t* = 0.204
			*p*=0.839
5 minutes	121 ± 6.9^ab^	125.18 ± 15^a^	*t* = 1.190
			*p*=0.241
10 minutes	115.4 ± 8.9^ac^	123.4 ± 16.5^a^	*t* = 1.873
			*p*=0.069
15 minutes	117.8 ± 8.9^ab^	125 ± 12.7^a^	*t* = 2.049
			*p*=0.047
30 minutes	115 ± 8.2^abcd^	125.5 ± 12.3a	*t* = 3.178
			*p*=0.003
45 minutes	114.3 ± 8^ac^	126 ± 11.6^a^	*t* = 3.701
			*p*=0.001
60 minutes	113.6 ± 8.5^acde^	125.7 ± 11.6^a^	*t* = 3.739
			*p*=0.001
Onset injection	111.7 ± 8.3^a^	123 ± 10.1^a^	*t* = 3.861
			*p* < 0.001
6 hours	120.5 ± 10.4^abg^	127.7 ± 7.7^b^	*t* = 2.486
			*p*=0.017
12 hours	123.7 ± 11.1^abfg^	130.9 ± 12.1	*t* = 1.951
			*p*=0.058
24 hours	122.3 ± 6.5^abefg^	129.5 ± 8.2^b^	*t* = 3.056
			*p*=0.004
Statistical test of repeated analysis of variance	*F* = 45.097	*F* = 7.912	
	*p* < 0.001	*p* < 0.001	

**Table 4:** Comparison of mean diastolic blood pressure of two groups at different times.

	Study group time		
	Control mean ± SD	Intervention mean ± SD	
Primary	84.3 ± 5.1	82.8 ± 7.2	*t* = 0.781
			*p*=0.440
5 minutes	75.1 ± 4.5^ab^	78.1 ± 10.1	*t* = 1.204
			*p*=0.236
10 minutes	71.6 ± 6.1^ac^	75.5 ± 8.6^a^	*t* = 1.647
			*p*=0.108
15 minutes	74.6 ± 5.4^a^	74.9 ± 6.8^a^	*t* = 0.154
			*p*=0.879
30 minutes	73.6 ± 6.4^a^	77.5 ± 6^ab^	*t* = 1.952
			*p*=0.058
45 minutes	70.8 ± 4.2^ac^	78.3 ± 5.4^abd^	*t* = 4.850
			*p* < 0.001
60 minutes	70.1 ± 3.8^ac^	78.2 ± 5.5^ab^	*t* = 5.406
			*p* < 0.001
Onset injection	70.3 ± 3.8^a^	5 ± 3.74^a^	*t* = 2.243
			*p*=0.031
6 hours	75.1 ± 5.6^a^	4.3 ± 7.77	*t* = 1.443
			*p*=0.159
12 hours	79 ± 7.5^bhfg^	80 ± 6.4^b^	*t* = 0.788
			*p*=0.435
24 hours	75.7 ± 5.4^a^	77.4 ± 4.2	*t* = 1.065
			*p*=0.294
Statistical test of repeated analysis of variance	*F* = 16.604	*F* = 6.197	
	*p* < 0.001	*p* < 0.001	

**Table 5:** Comparison of the average heart rate of two groups at different times.

	Study group time		
	Control mean ± SD	Intervention mean ± SD	Result of the independent *t* -test
Primary	88.9 ± 9.4^a^	83.2 ± 9.1	*t* = 1.926
			*p*=0.062
5 minutes	75.4 ± 6.2^a^	80.6 ± 10.8	*t* = 1.839
			*p*=0.074
10 minutes	71.6 ± 12.3^a^	78.4 ± 12.2	*t* = 1.766
			*p*=0.085
15 minutes	74.2 ± 6.4^a^	77.3 ± 9.7	*t* = 1.187
			*p*=0.243
30 minutes	73 ± 6.6^a^	77.2 ± 9.1	*t* = 1.640
			*p*=0.109
45 minutes	72.9 ± 7.2^a^	78.1 ± 8	*t* = 2.149
			*p*=0.038
60 minutes	71.5 ± 6.5^ab^	77.1 ± 8.8^a^	*t* = 2.277
			*p*=0.029
Onset injection	75.5 ± 6^a^	78.6 ± 7	*t* = 1.500
			*p*=0.142
6 hours	79 ± 6.3^adeg^	79 ± 6.7	*t* = 0.024
			*p*=0.981
12 hours	81.6 ± 9.6^ag^	81.4 ± 6.2	*t* = 0.058
			*p*=0.954
24 hours	79.5 ± 3.4^aefg^	80.6 ± 5.3	*t* = 0.746
			*p*=0.461
Statistical test of repeated analysis of variance	*F* = 15.429	*F* = 3.565	
	*p* < 0.001	*p*=0.006	

## Data Availability

The data used to support the findings of this study are available from the corresponding author upon request.
